# Mass Gatherings and Respiratory Disease Outbreaks in the United States – Should We Be Worried? Results from a Systematic Literature Review and Analysis of the National Outbreak Reporting System

**DOI:** 10.1371/journal.pone.0160378

**Published:** 2016-08-18

**Authors:** Jeanette J. Rainey, Tiffani Phelps, Jianrong Shi

**Affiliations:** Division of Global Migration and Quarantine, Centers for Disease Control and Prevention, Atlanta, GA, United States of America; Columbia University, UNITED STATES

## Abstract

**Background:**

Because mass gatherings create environments conducive for infectious disease transmission, public health officials may recommend postponing or canceling large gatherings during a moderate or severe pandemic. Despite these recommendations, limited empirical information exists on the frequency and characteristics of mass gathering-related respiratory disease outbreaks occurring in the United States.

**Methods:**

We conducted a systematic literature review to identify articles about mass gathering-related respiratory disease outbreaks occurring in the United States from 2005 to 2014. A standard form was used to abstract information from relevant articles identified from six medical, behavioral and social science literature databases. We also analyzed data from the National Outbreaks Reporting System (NORS), maintained by the Centers for Disease Control and Prevention since 2009, to estimate the frequency of mass gathering-related respiratory disease outbreaks reported to the system.

**Results:**

We identified 21 published articles describing 72 mass gathering-related respiratory disease outbreaks. Of these 72, 40 (56%) were associated with agriculture fairs and Influenza A H3N2v following probable swine exposure, and 25 (35%) with youth summer camps and pandemic Influenza A H1N1. Outbreaks of measles (n = 1) and mumps (n = 2) were linked to the international importation of disease. Between 2009 and 2013, 1,114 outbreaks were reported to NORS, including 96 respiratory disease outbreaks due to Legionella. None of these legionellosis outbreaks was linked to a mass gathering according to available data.

**Conclusion:**

Mass gathering-related respiratory disease outbreaks may be uncommon in the United States, but have been reported from fairs (zoonotic transmission) as well as at camps where participants have close social contact in communal housing. International importation can also be a contributing factor. NORS collects information on certain respiratory diseases and could serve as a platform to monitor mass gathering-related respiratory outbreaks in the future.

## Introduction

Mass gatherings create environments conducive to the transmission of infectious disease including pandemic influenza. [1–11]. Some mass gatherings such as outdoor sporting events may involve limited social mixing and are held in settings with ample ventilation. Other mass gatherings, however, can involve significant social mixing over several days such as professional conferences and music festivals [1–4]. Intensely crowded settings can lead to high secondary attack rates even when a circulating pathogen has a relatively low transmission probability [1–4]. Travelers to mass gatherings can not only introduce an infectious disease to a previously unaffected area, but can also amplify transmission at the gathering and further disseminate transmission following their return home. This was recently demonstrated by the propagation of the first wave of the 2009 H1N1 pandemic (pdm09H1N1) following a large Easter holiday gathering in Iztapalapa, Mexico [6]. Similar mass gatherings have been linked to the propagation of the Great Pandemic in 1918 and the Asian Flu Pandemic in 1957 [7–8].

During future influenza pandemics or other public health emergencies in the United States, state and local public health officials will have to consider modifying, postponing, or cancelling mass gatherings. This decision could depend on the severity of the pandemic as well as the timing, duration and size of the event and whether people will be traveling to and from the event from other (affected or not-yet-affected) communities [12]. If the risk of severe disease is low, then other non-pharmaceutical interventions could be recommended to minimize the potential for disease transmission. Despite these recommendations, limited empirical information exists on the frequency and characteristics of mass gathering-related respiratory disease outbreaks in the United States.

To address this gap, we conducted a systemic review of the published literature and analyzed the National Outbreak Reporting System (NORS) database maintained by the United States (U.S.) Centers for Disease Control and Prevention (CDC) since 2009 [13]. The objectives of this project included describing the frequency of mass gathering-related respiratory disease outbreaks occurring in the United States, highlighting the likely causes of these outbreaks, and documenting any patterns or shared characteristics across the identified outbreaks.

## Methods

### Data collection

#### Definitions

Mass gatherings can be defined as large events involving more than 1,000 persons in a specific location for a shared purpose [14]. We applied this definition in our literature review but also included camp sessions of at least 100 participants because the cumulative aggregation of participants over the camp season can easily exceed 1,000 persons. Because NORS does not include a specific field for mass gatherings, we analyzed data from the NORS database for outbreaks involving person-to-person transmission and transmission at camps, conferences, banquets, sporting events, and religious locations. Information on the setting for outbreaks involving other modes of transmission (e.g. water-borne) is not routinely reported to NORS. For all reported outbreaks, we used the number of persons exposed to approximate size of the mass gathering. We deferred to the author’s definition of outbreak in our literature review. Disease events reported to NORS were assumed to meet CDC’s outbreak definitions [13].

#### Systematic Literature Review

Six medical, behavioral, and social science literature databases were searched for relevant articles published from January 1, 2005 to December 31, 2014. These included Medline^®^ (National Library of Medicine) and five non-Medline databases: Embase^®^ (Excerpta Medica Database, Elsevier), Global Health, CAB Abstracts, Web of Science, and Scopus. A CDC reference librarian performed the initial search using the following strategy: ((mass OR public OR large OR general OR sport* OR community OR public) ADJ2 (gather* OR event* OR assembl* OR meeting*)) OR concert* OR theater* OR auditorium* OR amphitheater* OR Olympic* OR (world ADJ cup) OR festival* OR carnival* OR ((country OR county OR state OR world) ADJ fair*) OR world expo OR amusement park* OR cruise ship*AND Influenza OR pneumonia OR SARS OR flu OR H1N1 OR H3N2 OR coronavirus OR tuberculosis OR TB OR ((lung* OR respiratory) ADJ2 (infection* OR virus*)) OR MERS OR measles OR mumps OR acute respiratory syndrome OR legionnaires OR legionella OR infectious OR communicable OR illness OR disease* AND (transmission* OR outbreak OR epidemi* OR pandemic* OR cluster*) AND (at least one of the states in the United States).

To identify publications about outbreaks occurring at recreational, religious, cultural, and academic camps, the same search was repeated using the keywords (camp OR camping) AND Influenza OR pneumonia OR SARS OR flu OR H1N1 OR H3N2 OR coronavirus OR tuberculosis OR TB OR ((lung* OR respiratory) ADJ2 (infection* OR virus*)) OR MERS OR measles OR mumps OR acute respiratory syndrome OR legionnaires OR legionella OR infectious OR communicable OR illness OR disease* AND (transmission* OR outbreak OR epidemi* OR pandemic* OR cluster* OR spread) AND (at least one of the United States). We included “ADJ” as an adjacency operator to command the database to search for a word (or words) near another word within a certain number of words. For example, (mass ADJ2 outbreak) searched for the word “mass” within two words of the word “outbreak” picking up “mass outbreak” OR “outbreak during mass event”. The search query varied slightly across the six databases (S1 File).

Only articles written in English were eligible for inclusion. Duplicate articles identified during the initial database search were removed prior to aggregating results in an EndNote file. The titles and abstracts of the remaining articles were reviewed by one person and categorized as relevant if the title included one or a combination of the following terms: mass gathering (or a specific event or setting associated with mass gatherings) and outbreak (or a specific syndrome or infection). The article was also included if the abstract, if available, described information on the occurrence of an infectious disease or illness at a mass gathering (or specific event or setting associated with mass gatherings). Articles on enteric or skin infections were excluded.

We reviewed each article that met the criteria in full. If the manuscript addressed our project objectives, then we completed a standard abstraction form for the article title, publication date, mass gathering name, venue type, dates, location, approximate size, and pathogen involved as well as the number of probable and confirmed cases, age groups of cases, likely outbreak cause and response of mass gathering organizers. At a minimum, each article included in our final analysis described the following: 1) purpose of gathering, 2) month and year of gathering, 3) location or venue type (indoor or outdoor), 4) type of syndrome or infectious disease outbreak, 5) number of confirmed or probable cases, 6) information on the index case(s), and 7) possible cause(s) of the outbreak. If not mentioned in the article, the approximate size of the mass gathering was obtained by contacting the corresponding author or the mass gathering organizer. All project types were eligible for inclusion including outbreak investigation reports, case series, cross-sectional surveys, case-control studies, and intervention studies. No additional quality criteria for article inclusion were applied due to the variability in the study types [15]. Articles describing modeling studies, outbreaks on conveyances or at schools or businesses, policy papers, and outbreaks occurring before 2005 were excluded. We focused on mass gathering-related outbreaks occurring between 2005 and 2014 to provide a sufficiently long time frame to detect any trends or patterns before, during, and after the 2009 H1N1 pandemic while limiting results from the initial literature search criteria to a manageable size. Information from completed abstraction forms were entered into an Access database and imported into SAS for analysis.

#### National Outbreak Reporting System

NORS is a web-based national surveillance system for enteric waterborne, foodborne, person-to-person, animal associated, and environmental disease outbreaks as well as non-enteric waterborne outbreaks [13]. Health departments can voluntarily report outbreaks due to any other cause not mentioned above. NORS was fully deployed in 2009 and is maintained by CDC. We analyzed data from the NORS database for outbreaks involving person-to-person transmission that occurred between January 2009 and December 2013. Data describing water-borne outbreak were available through December 2012 only. Other available data included investigation methods, outbreak onset dates, number of exposed participants, total number of confirmed and probable cases, geographic location of the outbreak, and etiology, if known. Data were provided in an Access database and then imported into SAS (version 9.3, Cary, NC) for analysis.

### Analysis

We generated descriptive statistics on the frequency, type, size, venue, geographic spread and dates of mass gathering-related respiratory disease outbreaks identified from the literature review and NORS database. Additional qualitative analysis was performed on information abstracted from the literature review on the causes of the outbreaks. No personal identifying information on individual patients was collected.

### Ethics Approval

The project was reviewed by CDC’s Human Subjects Research Office and approved as a public health evaluation activity.

## Results

### Published literature

The literature database search resulted in 835 articles (475 and 360 using the general mass gathering and camping keywords, respectively) (Table 1). We identified 54 articles as relevant from the initial review of titles and abstracts and included 9 additional articles identified from the references of these 54 articles. Of these 63 total articles, 21 were included in our analysis following full review [16–36]. We excluded 42 articles primarily because the article did not report a respiratory disease outbreak (n = 18, 43%) or was not related to a mass gathering (n = 9, 21%).

**Table 1:** Categorization of the published literature reviewed for identifying mass gathering-related respiratory disease outbreaks in the United States, January 1, 2005–December 31, 2014.

	General Mass Gatherings	Camps	From References	Total
Literature database search results^a^	475	360	---	835
Relevant from title and abstract review	38	16	9	63
Included following full article review	7	11	3	21
Excluded articles after full review				
Duplicate	3	2	0	5
Not found	0	2	0	2
Not respiratory disease outbreak	17	1	0	18
Not mass gathering	4	0	5	9
Animal health	4	0	1	5
Not in United States	1	0	0	1
Experiment	2	0	0	2

^a^Databases included Medline^®^ (National Library of Medicine) and five non-Medline databases: Embase^®^ (Excerpta Medica Database, Elsevier), Global Health, CAB Abstracts, Web of Science, and Scopus.

The 21 articles included 18 manuscripts describing 14 individual mass gathering-related respiratory disease outbreaks (the same outbreak could be described in more than one article) and three articles [21, 25, 36] describing pathogen-specific summaries of outbreaks across different settings and included 59 additional mass gathering-related outbreaks (Table 2, S2 File). Thus, we identified 72 different mass gathering-related respiratory disease outbreaks. Of these, 1 occurred in 2005, 3 in 2007, 27 in 2009, 1 in 2011, 40 in 2012, and none in 2013 or 2014.

**Table 2:** Relevant articles about mass gathering-related respiratory disease outbreaks listed by year of outbreak and pathogen, identified from a literature review of articles published during 2005 and 2014.

Year^a^	Pathogen	Transmission	Type of Gathering	Location	Size of Gathering^b^	Length of Gathering (Days)	Estimated Number of Cases^c^	Age of Cases (Years)	Factors in outbreak	Reference
2005	Mumps	Person-to-person	Camp	New York	541	52	31	10–41	International importation of infectious case; Delayed diagnosis and reporting; Close social mixing and contact in communal housing/activities	CDC [16]; Schaffzin JK, et al. [17]
2007	Measles	Person-to-person	Sporting event	Pennsylvania	265,000	10	7^d^	12–53	International importation of infectious case; Contact with susceptible persons before and during travel and at event.	CDC [18]; Chen TH, et al. [19]
2007	Influenza A H1N1 (3SIV)	Animal (zoonotic)	Fair	Ohio	Unknown	Unknown	24	Unknown	Contact with swine	Killian ML, et al. [20]
2007	Influenza A H1N1 (3SIV)	Animal (zoonotic)	Fair	Multiple—Ohio^e^; Illinois; Michigan	Unknown	Unknown	4^f^	16 mos—48 yrs	Visiting near swine barn or contact with swine	Shinde V [21]
2009	Influenza A H1N1	Person-to-person	Camp	North Carolina	1,650^g^	56	67^h^	Unknown	Close social mixing/contact in communal housing	CDC [22]
2009	Influenza A H1N1	Person-to-person	Camp	North Carolina	700	7	52^i^	> 10	Close social mixing and contact in communal housing/activities	Doyle TJ, et al [23]
2009	Influenza A H1N1	Person-to-person	Camp	Louisiana	217	4^j^	59	5–69	Increased risk among participants with hematologic and oncologic conditions; Close social mixing and contact in communal housing/activities	Morrison C, et al. [24]
2009	Influenza A H1N1	Person-to-person	Camp	Multiple—Maine	Unknown	Unknown	19 outbreaks of influenza-like-illness and ≥3 confirmed cases	Unknown	Close social mixing and contact in communal housing/activities; Number of campers per session and per cabin	Robinson S, et al. [25]
2009	Influenza A H1N1	Person-to-person	Camp	Alabama	800^k^	28	15^k^	8–14^k^	Close social mixing and contact in communal housing/activities	Kimberlin D, et al. [26]
2009	Influenza A H1N1	Person-to-person	Camp	North Carolina	7,906^l^	73	119^m^	9–68	Close social mixing and contact in communal housing/activities including in classroom	Tsalik E, et al. [27]; Tsalik E, et al. [28]
2009	Influenza A H1N1	Person-to-person	Camp	Washington	145	6	60^n^	> 10	Close social mixing and contact in communal housing/activities	Sugimoto JD, et al. [29]
2009	Mumps	Person-to-person	Camp	New York	400	40	25^o^	9–30	International importation of infectious case; close social mixing and contact in communal housing/activities	CDC [30]; CDC [31]
2009	Influenza A H3N2 (SIV)	Animal (zoonotic)	Fair	Kansas	Unknown	Unknown	1	12	Contact with swine	Cox CH, et al. [32]
2011	Influenza A H3N2v	Animal (zoonotic)	Fair	Pennsylvania	~70,000	7	89^p^	Unknown	Contact with swine	CDC [33]; Wong KK, et al. [34]
2012	Influenza A H3N2v	Animal (zoonotic)	Fair	Indiana	Unknown	7	4	Unknown	Contact with swine	CDC [35]
2012	Influenza A H3N2v	Animal (zoonotic)	Fair	Multiple—Illinois; Indiana^e^; Maryland; Michigan; Minnesota; Ohio; Pennsylvania; Wisconsin	~21,000–1.7 million^q^	5–12^q^	From 1 to 73 (across 39 fairs reporting cases)	3 months—74 years	Visiting fair or contact with swine; Increased transmissibility of H3N2v (reassortant with M gene from pdm09H1N1)	Jhung MW, et al. [36]

^a^Reflects year during which initial outbreak-related case(s) were detected and reported.

^b^Estimated size of gathering obtained from article, personal communication with corresponding author or communication with the International Association of Fairs and Exhibitions (IAFE).

^c^Reflects case counts reported in the manuscript. Attack rates, when available, are reported in S2 File.

^d^Includes measles cases resulting from travel-related transmission.

^e^Indicates second reference to same outbreak.

^f^Includes only laboratory confirmed cases of Influenza A H1N1 (3SIV).

^g^Size and length of gathering combined across multiple-camps and 2 sessions.

^h^65 camp participants with influenza-like-illness across two camp sessions; 2 participants confirmed with Influenza A H1N1 during second session.

^i^Among 212 camp participants completing survey; 52 cases includes 49 camp participants (12 of whom were laboratory confirmed) and 3 identified from household contacts of ill camps participants.

^j^Camp closed early due to number of participants with influenza-like-illness and confirmation of 4 participants with Influenza A H1N1.

^k^Combined across 4 two-week sessions. In 3^rd^ session, 12 cases identified (including 4 confirmed with Influenza A H1N1). In 4^th^ session, 3 cases confirmed with Influenza A H1N1; cases aged 8 to 14 years.

^l^Numerous academic and recreational camps on university campus with length varying from 3 to 73 days, all camps closed early due to identified illnesses.

^m^Across two clusters; first involving 3 camps and 64 cases and second involving 2 camps and 55 cases.

^n^Includes 49 camp participants (from 96 campers responding to survey) and 11 additional cases among household contacts (of 136 household contacts from 41 households with an ill camper). Five camp participants confirmed with Influenza A H1N1.

^o^Outbreak spread to other counties in New York and New Jersey for total of 1,523 mumps cases.

^p^Includes 3 confirmed, 4 probable, and 82 suspected cases of Influenza A H3N2v.

^q^Estimates from IAFE based on available data from subset of fairs reporting Influenza A H3N2v cases; at time of project, no standards existed for calculating fair attendance.

#### Fairs

More than half (n = 44, 61%) of the 72 identified outbreaks occurred at state or county agricultural fairs (Table 3). All of these fairs were large multiday events ranging between 5 to 12 days with a mean daily attendance of 33,000 persons (personal communication on subset of fairs for which data were available: Marla Calico, International Association of Fairs and Exhibitions). Forty outbreaks were reported between 2011 and 2012 and involved transmission of Influenza A H3N2v at fairs across 9 states. The largest outbreak occurred at an agricultural fair in Indiana and resulted in 73 confirmed and probable cases. In this, as well as other H3N2v outbreaks, the majority of case-patients reported close contact with swine at the agricultural fairs, though additional but limited secondary person-to-person transmission was detected [33–36]. Children and adolescents less than 18 years of age represented the largest percentage of case-patients (92%) during these outbreaks [36]. Three outbreaks of Influenza A H1N1 (3SIV) occurred at agricultural fairs in 2007 in Ohio, Illinois, and Michigan [20–21]. The swine barn housed 235 pigs belonging to 133 exhibitors and was closed as a result of the outbreak in Ohio [20]. A single case of Influenza A H3N2 (3SIV) was detected in a 12-year old boy after attending an agricultural fair in Kansas in 2009.

**Table 3:** Frequency of mass gathering-related respiratory disease outbreaks identified from the published literature^a^ by setting, pathogen, and mode of transmission, January 1, 2005–December 31, 2014.

Setting	Pathogen	Mode of Transmission	Outbreak Frequency
Fairs	Influenza A H3N2v	Zoonotic^b^	40
	Influenza A H1N1 (3SIV)	Zoonotic	3
	Influenza A H3N2 (SIV)	Zoonotic	1
Camps	Influenza A H1N1	Person-to-person	25
	Mumps	Person-to-person	2
Sporting events	Measles	Person-to-person	1
Total			72

^a^From 21 reviewed articles.

^b^Minimal person-to-person transmission detected.

#### Camps

Twenty-seven (38%) of the 72 identified outbreaks occurred at residential recreational, academic, or cultural camps. Each lasted for three days or more and involved either Influenza A (H1N1) (n = 25) [22–29] or mumps (n = 2) [16–17, 30–31]. The size of most camp sessions was small, ranging between 100 to 600 campers and staff. Nineteen (76%) of the 25 Influenza A H1N1 (pdm09H1N1) outbreaks were reported from an online survey of 2009 summer camp participants in Maine [25]. In this survey, camp-related outbreaks (defined as having at least three confirmed cases of pdm09H1N1) were statistically associated with having both a larger number of camp participants per session and a larger number of campers per cabin. The six other camp-related pdm09H1N1 outbreaks occurred in North Carolina (n = 3) [22–23, 27–28], Louisiana (n = 1) [24], Alabama (n = 1) [26], and Washington State (n = 1) [29]. One outbreak involved 119 cases occurring across two clusters in a multi-camp setting at a North Carolina university with more than 7,900 participants [32–33]. The authors found that this transmission occurred rapidly during academic camps and was likely due to close and frequent contact during classroom time and social mixing outside of class. Two camps (the university camp in North Carolina [27–28] and a camp for children with hematological and oncological conditions in Louisiana [24]) closed early due to the large number of participants presenting influenza-like-illness (ILI).

Two mumps outbreaks were identified in this review—both occurred at a camp for boys in upstate New York following international importations of infectious mumps cases from the United Kingdom. The first outbreak took place in 2007 and resulted in 31 cases among 541 campers and staff; the staff represented 60% of all case-patients [16–17]. The second occurred in 2009 and caused 25 cases among 400 campers and staff [30–31]. In both outbreaks, transmission was associated with prolonged close contact in communal housing and camp activities despite a two-dose MMR vaccination coverage > 85% among school-aged campers. A delay in mumps diagnosis also likely contributed to the 2007 outbreak. During the 2007 outbreak, camp activities were either cancelled or postponed [16–17]. These outbreaks resulted in substantial community transmission when campers returned home—more than 1,500 community cases (including 19 hospitalizations) occurred in New York and New Jersey in 2009 [30–31].

#### Sporting events

The final mass gathering-related outbreak identified in this review involved measles transmission at an international youth sporting event in Pennsylvania [18–19]. The youth sporting event outbreak was caused by the international importation of measles in a participant from Japan and resulted in six additional cases of measles. The index case from Japan infected three others before or during travel to Pennsylvania and a fourth case at the sporting event. The remaining two cases subsequently contracted measles in another state from the case-patient infected at the sporting event. Public health officials worked with event organizers to provide post-exposure measles-mumps-rubella (MMR) vaccination to close contacts of case-patients without documentation of at least two doses of measles vaccine or who were negative for measles IgG antibody [19].

Four of the 34 articles excluded from our analysis described health events at six large mass gatherings in Arizona between 2008 and 2011 [37], at Kansas Speedway NASCAR racing events between 2007 and 2010 [38], at the annual New York State Fair between 2004 and 2008 [39] and at a large (>40,000 participants) summer camp in Virginia in 2005 [40]. Analysis of syndromic surveillance data and/or onsite clinic records maintained at these gatherings identified heat or dehydration-related illnesses, injuries, and enteric infections as the most frequently reported conditions at these mass gatherings; no infectious respiratory disease outbreaks were detected. Additionally, no single-day mass gathering-related outbreaks were identified in our review.

### National Outbreak Reporting System

Between 2009 and 2013, 1,114 outbreaks were reported to NORS (Table 4). This included 96 infectious respiratory disease outbreaks due to Legionella—all of which were reported as water-borne outbreaks (Legionella is not easily transmitted between human hosts). None of these outbreaks was linked to mass gatherings in our analysis. No other respiratory disease outbreaks were reported to NORS.

**Table 4:** Outbreaks reported to CDC’s National Outbreak Reporting System (NORS), 2009–2013, by year and mode of transmission.

Mode of Transmission	2009 (%)	2010 (%)	2011 (%)	2012 (%)	2013 (%)	Total (%)
** Foodborne**	118 (62)	148 (58)	133 (61)	142 (58)	154 (74)	695 (62)
**Waterborne**^a^	51 (27)	78 (31)	66 (30)	71 (29)	---	266 (24)
**Person-to-person**^b^	10 (5)	17 (7)	6 (3)	16 (7)	25 (12)	74 (7)
** Animal contact**	10 (5)	12 (5)	13 (6)	14 (6)	15 (7)	64 (6)
** Environmental**	1 (<1)	0 (0)	0 (0)	0 (0)	0 (0)	1 (<1)
** Unknown/indeterminate**	0 (0)	0 (0)	0 (0)	0 (0)	14 (7)	14 (1)
**Total**	190	255	218	243	208	1,114

^a^At time of analysis, 2013 waterborne disease outbreak data were not available. Waterborne transmission includes 96 legionellosis outbreaks; none were linked to mass gatherings.

^b^Person-to-person outbreaks (n = 74) were due to Norovirus (45), *Escherichia* (3), *Salmonella* (1), *Shigella* (1), and *Campylobacter* (1). The remaining 23 outbreak reports were missing etiology information.

## Discussion

To the best of our knowledge, this is the first effort to describe the frequency and characteristics of mass gathering-related respiratory disease outbreaks occurring in the United States. Mass gathering-related respiratory disease outbreaks appeared to be relatively rare during our 10-year project period, but were reported from agricultural fairs (zoonotic transmission) and summer camps where participants had close social contact in communal housing. Though legionellosis outbreaks were more common, none of these water-borne outbreaks was linked to a mass gathering in our analysis. The type and duration (multi-day events with communal housing) of mass gatherings could be important factors in mass gathering-related respiratory disease outbreaks. International participation may be a contributing factor for certain diseases.

As anticipated, we identified several mass gathering-related pdm09H1N1 outbreaks involving school-aged children [41]. These persons are a high-risk age group for the novel 2009 influenza virus. Compared to other settings, influenza transmission among children and teenagers is more likely to occur within households, school classes, peer groups, and sports teams [42–44]—all of these social mixing opportunities are prominent at summer camps. Children and adolescents can easily have more than 20 close contacts with other children lasting 5 minutes of more during a typical school day [45]. Both the number and duration of close contacts could be higher in residential camps. This is supported by the outbreaks reported from Maine where the camp size and the number of campers per cabin were associated with influenza outbreaks [25]. Though respiratory disease transmission can occur at summer camps, influenza transmission during the summer (in the United States) outside a pandemic is uncommon; pdm09HIN1 was circulating since early spring and was involved a number of school-based outbreaks in states reporting camp-related influenza outbreaks in summer 2009. No respiratory illnesses were detected from a syndromic surveillance system implemented at a large school-aged summer camp in Virginia in 2005 [40].

The majority of the other mass gathering-related respiratory disease outbreaks identified in this project occurred at agricultural fairs and was caused by Influenza A H3N2v. Prior to 2011–2012, human Influenza A (SIV) infections were rare; however, the H3N2v outbreaks resulted from the reassortment of swine Influenza A H3N2 virus and Influenza A pdm09H1N1 in the swine population, raising concerns about sustained human-to-human transmission and increased risk in the pediatric population [46]. Though only minimal human-to-human transmission of H3N2v was detected, close and repeated contact between humans and livestock at the estimated 2,380 agricultural fairs (personal communication: Marla J. Calico, International Association of Fairs and Exhibitions) that take place each year in the United States could create future opportunities for zoonotic transmission of influenza viruses as well as other possible respiratory infections [34, 36]. The syndromic surveillance system using emergency department chief complaint data was analyzed to detect the initial H3N2v outbreak in Indiana (personal communication: Shawn Richards, Indiana State Department of Health). This highlights the usefulness of such systems to rapidly detect temporal or spatial clusters of unusual health events associated with fairs and other mass gatherings.

The mumps and measles outbreaks demonstrate the importance of close collaboration between public health officials and mass gathering organizers to assess health risks to participants. As recommended in the articles, camp and sporting event organizers should verify immunization histories of all registered participants and team members [17, 31]. This is particularly important for measles, which is highly contagious through aerosolized viral particles; other control and prevention measures are likely to be ineffective. Organizers should also obtain information on disease transmission in the home countries of participants. These pre-gathering activities can help with the early case detection and outbreak prevention. The 2007 mumps outbreak, for example, was attributed to the international importation of mumps in a staff member followed by the delayed recognition of suspected mumps cases and prolonged and close contact between campers in communal housing [16–17].

NORS was developed to capture outbreaks related to enteric infections such as Norovirus, Shigella, and Salmonella as well as non-enteric waterborne diseases such as Legionnaires—an infection caused by a bacterium found naturally in the environment [47]. Though we could use exposure setting (e.g., camps, conference, etc.) as a proxy for mass gathering, this exposure setting was only reported for outbreaks involving person-to-person transmission. The 96 reported legionellosis outbreaks were unrelated to mass gatherings according to available data. A previous review of environmental and waterborne outbreaks reported to NORS between 2011 and 2012 indicated that the 4 (22%) of the 18 legionellosis outbreaks occurred at hotels; however, there was no additional information to determine on whether visitors or detected cases were involved in hotel-based conferences or other mass gatherings [48].

No other mass gathering outbreak due to pdm09H1N1 or other respiratory diseases was identified in this project including at large mass gatherings in Arizona, Kansas, New York, and Virginia [37–40]. This is consistent with previous assessments of international mass gatherings such as the Olympics that detected only marginal increases above baseline in the incidence of all infectious diseases including influenza with infections primarily limited to competitors and staff [5, 47]. Though we assume that all mass gatherings could increase the transmission of respiratory diseases, this may not be the case for diseases transmitted through infectious droplets where the probability of close and adequate contact (i.e., < 6 feet for influenza) with an infectious person may be too low for efficient transmission [49]. Even at large mass gatherings involving out-of-town visitors such as the May 2009 convocation speech in Arizona (during which no respiratory disease outbreaks were detected) [37], most participants likely stayed at hotels or at other low-density accommodations rather than in cabins or at campgrounds [5, 49]. In addition, many of these gatherings have well organized and pre-assigned seating. This could further reduce the ‘crowdedness’ and likelihood of close and prolonged contact with infectious persons [5]. This can be contrasted to the type of communal housing and social mixing associated with camp outbreaks described here. Variability in susceptibility among attendees as well as in the timing of the gathering in relation to local respiratory disease activity also likely play a role in transmission [9].

Our findings have several limitations. First, only a small percentage of outbreaks are likely reported in the literature. We used a broad database search criteria to minimize this limitation and identify all possible articles describing mass gathering-related respiratory disease outbreaks. Second, some outbreaks may not be identified as outbreaks. Active or passive surveillance likely influences the number of infectious respiratory disease outbreaks identified and reported. For example, active surveillance for variants of influenza viruses at fairs following the initial H3N2v outbreak in 2011 could have increased the number of outbreaks detected from this setting. In addition, while multiday residential-based mass gatherings can facilitate transmission, these settings may also help identify cases and an emerging outbreak through close monitoring of known participants. In non-residential settings, other non-disease related factors including crowd mood, age, weather, and alcohol and drug use could influence the use of illness-related medical services. These factors could further increase the variability of health event reporting [14]. Third, some respiratory diseases with longer incubation periods and many subclinical infections are unlikely to be identified during the mass gathering. Symptoms could appear once the participants have returned home and are then rarely linked or reported to the appropriate surveillance system [9, 49].

Advances in technology such as crowdsourcing applications (e.g. Flu Near You at URL: https://flunearyou.org and GermTrax at URL: http://germtrax.com) [50] as well as an increased use of social media along with real-time outbreak and syndromic event reporting could help address some of the surveillance limitations at mass gatherings. In Brazil, for example, a smart phone application was developed allowing persons attending the 2014 FIFA World Cup to provide information on their daily health status. This information was automatically aggregated into reports that were reviewed daily to identify potential infectious disease clusters or outbreaks [51]. Monitoring social media including Twitter was used to identify potential health issues and communicate accurate information about disease transmission during the 2012 London Olympics [52]. These approaches can complement other surveillance systems such as collection and analysis of syndromic event data from emergency department visits or onsite medical clinics [37]. Integration of these various platforms through a web-based system could allow public health officials to rapidly detect unusual disease activity [10, 53] even at smaller mass gatherings [54–55]. Recent research has also focused on the use of radio frequency identification devices [56–57] as well as video analysis technology [58] to capture social mixing and contact patterns at mass gatherings. These contact data can be used to simulate or model the transmission of various infectious disease pathogens including pandemic influenza. Simulations or mathematical models can help describe the risk of potential outbreaks and highlight where and when enhanced disease transmission mitigation at mass gatherings should be considered.

Finally, NORS was developed primarily to capture outbreaks related to enteric infections as well as non-enteric waterborne diseases such as Legionnaires. Voluntary reporting of other types outbreaks likely impacted the probability of identifying mass gathering related respiratory disease outbreaks from the NORS database. However, because state and local health departments are familiar with this system, NORS could incorporate standard mass gathering and additional outbreak definitions and serve as a platform to monitor mass gathering-related respiratory outbreaks in the future.

## Conclusion

We identified a relatively small number of mass gathering-related respiratory disease outbreaks occurring in the United States between 2005 and 2014. This could suggest a low risk of respiratory disease transmission at most types of gatherings—even during a pandemic. However, because not all outbreaks are reported in the published literature or to NORS, a follow-up state and local health department assessment on the number and characteristics of mass gathering-related respiratory disease outbreaks could help validate or complement the findings reported here. Additional research to strengthen existing surveillance approaches to detect outbreaks and better quantify the social mixing patterns at different mass gatherings could provide additional insight into the types of mass gatherings at greatest risk of respiratory disease outbreaks.

## Supporting Information

S1 File Literature database search strategy. (PDF)

S2 File Summary information from articles included in systematic literature review. (PDF)

## References

[1] Abubakar I, Gautret P, Brunette GW, Blumberg L, Johnson D, Poumerol G, et al Global perspectives for prevention of infectious diseases associated with mass gatherings. *Lancet Infect Dis* 2012; 12:66–74. 10.1016/S1473-3099(11)70246-8 22192131

[2] Chowell G, Nishiura H, Viboud C. Modeling rapidly disseminating infectious disease during mass gatherings. *BMC Medicine* 2012 10:159 10.1186/1741-7015-10-159 23217051 PMC3532170

[3] Johansson A, Batty M, Hayashi K, Al Bar O, Marcozzi D, Memish ZA. Crowd and environmental management during mass gatherings. *Lancet Infect Dis* 2012;12:150–6. 10.1016/S1473-3099(11)70287-0 22252150

[4] Rashid H, Haworth E, Shafi S, Memish ZA, Booy R. Pandemic influenza: mass gatherings and mass infection. *Lancet Infect Dis* 2008; 8:526–7. 10.1016/S1473-3099(08)70186-5 18684671 PMC7128517

[5] Ishola DA, Phin N. Could influenza transmission be reduced by restricting mass gatherings? Towards an evidence-based policy framework. *J Epidmiol Globl Health* 2011;1:30–60. 10.1016/j.jegh.2011.06.004 PMC7104184 23856374

[6] Zepeda-Lopez HM, Perea-Araujo L, Miliar-García A, Dominguez-López A, Xoconostle-Cázarez B, et al Inside the outbreak of the 2009 influenza A (H1N1)v virus in Mexico. *PLoS One* 2010;5:e13256 10.1371/journal.pone.0013256 20949040 PMC2951908

[7] United States Department of Health and Human Services. The Great Pandemic. Available from URL: http://www.flu.gov/pandemic/history/1918/your_state/northeast/Pennsylvania/. Accessed on September 22, 2015.

[8] Langmuir AD. Asian influenza in the United States. *Ann Intern Med* 1958;49:483–92. 13571835 10.7326/0003-4819-49-3-483

[9] Chowell G, Echevarría-Zuno S, Viboud C, Simonsen L, Tamerius J, Miller MA, et al Characterizing the epidemiology of the 2009 influenza A/H1N1 pandemic in Mexico. *PLoS Med* 2011 8:e1000436 10.1371/journal.pmed.1000436 21629683 PMC3101203

[10] Khan K, McNabb SJ, Memish ZA, Eckhardt R, Hu W, Kossowsky D, et al Infectious disease surveillance and modelling across geographic frontiers and scientific specialties. *Lancet Infect Dis* 2012;12:222–30. 10.1016/S1473-3099(11)70313-9 22252149

[11] Shi P, Keskinocak P, Swann JL, Lee BY. The impact of mass gatherings and holiday traveling on the course of an influenza pandemic: a computational model. *BMC Public Health* 2010;10:778 10.1186/1471-2458-10-778 21176155 PMC3022852

[12] Centers for Disease Control and Prevention (CDC). Interim CDC guidance for public gatherings in response to human infections with novel influenza A (H1N1), 2009. Available from URL: http://www.cdc.gov/h1n1flu/guidance/public_gatherings.htm. Last accessed on March 26, 2015.

[13] Centers for Disease Control and Prevention (CDC). Notification of Outbreak Reporting System. Available from URL: http://www.cdc.gov/nors/about.html. Last accessed on October 1, 2015.

[14] Milsten AM, Maguire BJ, Seaman KG. Mass-gathering medical care: a review of the literature. *Prehosp Disaster Med* 2002;17:151–62. 12627919 10.1017/s1049023x00000388

[15] Moher D, Liberati A, Tetzlaff J, Altman DG. Preferred reporting items for systematic reviews and meta-analyses: the PRISMA statement. *PloS Med* 2009;6:e1000097 10.1371/journal.pmed.1000097 19621072 PMC2707599

[16] Centers for Disease Control and Prevention (CDC). Mumps outbreak at a summer camp—New York, 2005. *MMWR—Morbidity & Mortality Weekly Report* 2006;55:175–7. 16498381

[17] Schaffzin JK, Pollock L, Schulte C, Henry K, Dayan G, Blog D, et al Effectiveness of previous mumps vaccination during a summer camp outbreak. *Pediatrics* 2007;120:e862–e868. 17908742 10.1542/peds.2006-3451

[18] Centers for Disease Control and Prevention (CDC). Multistate measles outbreak associated with an international youth sporting event—Pennsylvania, Michigan, and Texas, August-September 2007. *MMWR—Morbidity & Mortality Weekly Report* 2008;57:167–73. 18288074

[19] Chen TH, Kutty P, Lowe LE, Hunt EA, Blostein J, Espinoza R, et al Measles outbreak associated with an international youth sporting event in the United States, 2007. *Pediatric Infectious Disease Journal* 2010;29:794–800. 10.1097/INF.0b013e3181dbaacf 20400927

[20] Killian ML, Swenson SL, Vincent AL, Landgraf JG, Shu B, Lindstrom S, et al Simultaneous infection of pigs and people with triple-reassortant swine influenza virus H1N1 at a U.S. county fair. *Zoonoses & Public Health* 2013;60:196–201. 22776714 10.1111/j.1863-2378.2012.01508.x

[21] Shinde V, Bridges CB, Uyeki TM, Shu B, Balish A, Xu X, et al Triple-Reassortant Swine Influenza A (H1) in Humans in the United States, 2005–2009. *NEJM* 2009;360:2616–25. 10.1056/NEJMoa0903812 19423871

[22] Centers for Disease Control and Prevention. Oseltamivir-resistant 2009 pandemic influenza A (H1N1) virus infection in two summer campers receiving prophylaxis—North Carolina, 2009. *MMWR—Morbidity & Mortality Weekly Report* 2009;58:969–72. 19745803

[23] Doyle TJ, Hopkins RS. Low secondary transmission of 2009 pandemic influenza A (H1N1) in households following an outbreak at a summer camp: relationship to timing of exposure. *Epidemiology and Infection* 2011;139:45–51. 10.1017/S095026881000141X 20561391

[24] Morrison C, Maurtua-Neumann P, Myint MT, Drury SS, Begue RE. Pandemic (H1N1) 2009 outbreak at camp for children with hematologic and oncologic conditions. *Emerg Infect Dis* 2011;17:879. 10.3201/eid1701.091499 PMC3204616 21192861

[25] Robinson S, Averhoff F, Kiel J, Blaisdell L, Haber M, Sites A, Copeland D. Pandemic influenza A in residential summer camps—Maine, 2009. *Pediatric Infectious Disease Journal* 2012;31:547–550. 10.1097/INF.0b013e31824f8124 22414902

[26] Kimberlin DW, Escude J, Gantner J, Ott J, Dronet M, Stewart TA, et al Targeted antiviral prophylaxis with Oseltamivir in a summer camp setting. *Archives of Pediatrics & Adolescent Medicine* 2010;164:323–327. 20124132 10.1001/archpediatr.2009.299

[27] Tsalik EL, Cunningham CK, Cunningham HM, Lopez-Marti MG, Sangvai DG, Purdy WK, et al An Infection Control Program for a 2009 influenza A H1N1 outbreak in a university-based summer camp. *Journal of American College Health* 2011;59:419–26. 21500062 10.1080/07448481.2010.534215

[28] Tsalik EL, Hendershot EF, Sangvai DG, Cunningham HM, Cunningham CK, Lopez-Marti MG, et al *Journal of Clinical Virology* 2010;47:286–288. 20064740 10.1016/j.jcv.2009.12.012

[29] Sugimoto JD, Borse NN, Ta ML, Stockman LJ, Fischer GE, Yang Y, et al The effect of age on transmission of 2009 pandemic influenza A (H1N1) in a camp and associated households. *Epidemiology* 2011;22:180–187. 10.1097/EDE.0b013e3182060ca5 21233714 PMC3755879

[30] Centers for Disease Control and Prevention (CDC). Mumps Outbreak—New York, New Jersey, Quebec, 2009. *MMWR—Morbidity & Mortality Weekly Report* 2009;58:1270–4. 19940833

[31] Centers for Disease Control and Prevention (CDC). Update: mumps outbreak—New York and New Jersey, June 2009-January 2010. *MMWR—Morbidity & Mortality Weekly Report* 2010;59:125–9. 20150887

[32] Cox CH, Neises D, Garten RJ, Bryant B, Hesse RA, Anderson GA, et al Swine influenza virus A (H3N2) infection in human, Kansas, USA, 2009. *Emerg Infect Dis* 2011;7:1143–4. 10.3201/eid1706.101488 PMC3358206 21749798

[33] Centers for Disease Control and Prevention (CDC). Swine-Origin Influenza A (H3N2) Virus Infection in Two Children—Indiana and Pennsylvania, July–August 2011. *MMWR—Morbidity & Mortality Weekly Report* 2011;60:1213–5. 21900876

[34] Wong KK, Greenbaum A, Moll ME, Lando J, Moore EL, Ganatra R et al Outbreak of influenza A (H3N2) variant virus infection among attendees of an agricultural fair, Pennsylvania, USA, 2011. *Emerg Infect Dis* 2012;18:1937–44 10.3201/eid1812.121097 23171635 PMC3557885

[35] Centers for Disease Control and Prevention (CDC). Notes from the field: Outbreak of influenza A (H3N2) virus among persons and swine at a county fair—Indiana, July 2009. *MMWR—Morbidity & Mortality Weekly Report* 2012;61:561. 22832938

[36] Jhung MA, Epperson S, Biggerstaff M, Allen D, Balish A, Barnes N, et al. Outbreak of Variant Influenza A(H3N2) Virus in the United States. *Clinical Infectious Diseases* 2013:12: 1703–1712. 10.1093/cid/cit649 PMC5733625 24065322

[37] Pogreba-Brown K, McKeown K, Santana S, Diggs A, Stewart J, Harris RB. Public Health in the Field and the Emergency Operations Center: Methods for Implementing Real-Time Onsite Syndromic Surveillance at Large Public Events. *Disaster Medicine and Public Health Preparedness* 2013;7:467–74. 10.1017/dmp.2013.83 24274126

[38] Selig B, Hastings M, Cannon C, Allin D, Klaus S, Diaz FJ. Effect of Weather on Medical Patient Volume at Kansas Speedway Mass Gatherings. *J Emerg Nurs* 2013;39:e39–44. 10.1016/j.jen.2011.10.002 22204886

[39] Grant WD, Nacca NE, Prince LA, Scott JM. Mass-gathering medical care: retrospective analysis of patient presentations over five years at a multi-day mass gathering. *Prehosp Disaster Med* 2010;25:183–7. 20468001 10.1017/s1049023x00007950

[40] Centers for Disease Control and Prevention (CDC). Surveillance for early detection of disease outbreaks at an outdoor mass gathering—Virginia, 2005. *MMWR—Morb Mortal Wkly Rep* 2006;55:71–4. 16437057

[41] Shrestha SS, Swerdlow DL, Borse RH, Prabhu VS, Finelli L, Atkins CY, et al Estimating the burden of 2009 pandemic influenza A (H1N1) in the United States (April 2009-April 2010). *Clin Infect Dis* 2011;52(Suppl 1):S75–82. 10.1093/cid/ciq012 21342903

[42] Glezen WP, Couch RB. Interpandemic influenza in the Houston area, 1974–76. *N Engl J Med* 1978;298:587–592. 628375 10.1056/NEJM197803162981103

[43] Viboud C, Boelle PY, Cauchemez S, Lavenu A, Valleron AJ, Flahault A, et al Risk factors of influenza transmission in households. *Br J Gen Pract* 2004;54:684–689. 15353055 PMC1326070

[44] Glass LM, Glass RJ. Social contact networks for the spread of pandemic influenza in children and teenagers. *BMC Public Health* 2008;8:61 10.1186/1471-2458-8-61 18275603 PMC2277389

[45] Guclu H, Read J, Vukotich CJ Jr, Galloway D, Gao H, Rainey J, et al Social Contact Networks and Mixing Among Students in K-12 Schools in Pittsburgh, PA. *PLoS One* 2016;11:e0151139 10.1371/journal.pone.0151139 26978780 PMC4792376

[46] Chou YY, Albrecht RA, Pica N, Lowen AC, Richt JA, Garcia-Sastre A, et al The M Segment of the 2009 new pandemic H1N1 influenza virus is critical for its high transmission efficiency in guinea pig model. *J Virol* 2011;85:11235–41. 10.1128/JVI.05794-11 21880744 PMC3194962

[47] Centers for Disease Control and Prevention (CDC). Legionella (Legionnaires and Pontiac Fever)–Causes and Transmission. Available from URL: http://www.cdc.gov/legionella/about/causes-transmission.html. Last accessed on November 20, 2015.

[48] Beer KD, Gargano JW, Roberts VA, Reses HE, Hill VR, Garrison LE, et al Outbreaks Associated with Environmental and Undetermined Water Exposures—United States, 2011–2012. *MMWR—Morb Mortal Wkly Rep* 2015;64:849–51. 26270060 10.15585/mmwr.mm6431a3 PMC4584590

[49] Zieliński A. Evidence for excessive incidence of infectious diseases at mass gatherings with special reference to sporting events. *Przegl Epidemiol* 2009;63:343–51. 19899589

[50] Nsoesi EO, Kluberg SA, Mekaru SR, Majumder MS, Khan K, Hay SI, et al New digital technologies for the surveillance of infectious diseases at mass gathering events. *Clin Microbiol Infect* 2015;21:134–40. 10.1016/j.cmi.2014.12.017 25636385 PMC4332877

[51] Libel M. The world’s first application of participatory surveillance at a mass gathering: FIFA World Cup 2014. Available from URL: http://imed.isid.org/symposia.shtml. 10.2196/publichealth.7313 PMC5438444 28473308

[52] McCloskey B, Endericks T, Catchpole M, Zambon M, McLauchlin J, Shetty N, et al London 2012 Olympic and Paralympic Games: public health surveillance and epidemiology. *Lancet* 2014;383:2083–9. 10.1016/S0140-6736(13)62342-9 24857700 PMC7138022

[53] Gesteland PH, Gardner RM, Tsui FC, Espino KU, Rolfs RT, James BC, et al Automated syndromic surveillance for the 2002 Winter Olympics. *J Am Med Inform Assoc* 2003;10:547–54. 12925547 10.1197/jamia.M1352 PMC264432

[54] Sniegoski C, Loschen W, Dearth S, Gibson J, Lombardo J, Wade M, et al Super Bowl surveillance a practical exercise in multi-jurisdictional data sharing. *Adv Dis Surveill* 2007;4195.

[55] Polkinghorne BG, Massey PD, Durrheim DN, Byrnes T, MacIntyre CR. Prevention and surveillance of public health risks during extended mass gatherings in rural areas: the experience of the Tamworth Country Music Festival. *Aust Public Health* 2013;127:32–8. 10.1016/j.puhe.2012.09.014 23141111

[56] Stehlé J, Voirin N, Barrat A, Cattuto C, Colizza V, Isella L, et al Simulation of an SEIR infectious disease model on the dynamic contact network of conference attendees. *BMC Med* 2011;9:1–15. 21771290 10.1186/1741-7015-9-87 PMC3162551

[57] Isella L, Stehle J, Barrat A, Cattuto C, Pinton JF, Van den Broeck W. What’s in a crowd? Analysis of face-to-face behavioral networks. *Journal of Theoretical Biology* 2011;271:166–180. 10.1016/j.jtbi.2010.11.033 21130777

[58] Rainey JJ, Cheriyadat A, Radke RJ, Suzuki Crumly J, Koch DB. Estimating contact rates at a mass gathering by using video analysis: A proof-of-concept project. *BMC Pub Health* 2014;14:1101. 25341363 10.1186/1471-2458-14-1101 PMC4223750

