# Supplementary material for: Mass Gatherings and Respiratory Disease Outbreaks in the United States – Should We Be Worried? Results from a Systematic Literature Review and Analysis of the National Outbreak Reporting System
Source: PLoS One. 2016 Aug 18;11(8):e0160378. doi: 10.1371/journal.pone.0160378 (PMC4990208; doi:10.1371/journal.pone.0160378)
Supplement: S1 File — (PDF) [file pone.0160378.s001.pdf]

Supporting Information 1 – Literature database search strategy

Search Query: Infectious Outbreaks Mass Gatherings

Database	Strategy
**Medline (OVID); Embase (OVID); Global Health (OVID); CAB Abstracts (OVID)**	((mass OR public OR large OR general OR sport* OR community OR public) ADJ2 (gather* OR event* OR assembl* OR meeting*)) OR concert* OR theater* OR auditorium* OR amphitheater* OR Olympic* OR (world ADJ cup) OR festival* OR carnival* OR ((country OR county OR state OR world) ADJ fair*) OR world expo OR amusement park* OR cruise ship*<br><br>AND<br><br>Influenza OR pneumonia OR SARS OR flu OR H1N1 OR H3N2 OR coronavirus OR tuberculosis OR TB OR ((lung* OR respiratory) ADJ2 (infection* OR virus*)) OR MERS OR measles OR mumps OR acute respiratory syndrome OR legionnaires OR legionella OR infectious OR communicable OR illness OR disease*<br><br>AND<br><br>Transmission* OR outbreak OR epidemi* OR pandemic* OR cluster*<br><br>AND<br><br>(exp United States/ OR United States OR USA OR Alabama OR Alaska OR Arizona OR Arkansas OR California OR Colorado OR Connecticut OR Delaware OR Florida OR Georgia OR Hawaii OR Idaho OR Illinois OR Indiana OR Iowa OR Kansas OR Kentucky OR Louisiana OR Maine OR Maryland OR Massachusetts OR Michigan OR Minnesota OR Mississippi OR Missouri OR Montana OR Nebraska OR Nevada OR New Hampshire OR New Jersey OR New Mexico OR New York OR North Carolina OR North Dakota OR Ohio OR Oklahoma OR Oregon OR Pennsylvania OR Rhode Island OR South Carolina OR South Dakota OR Tennessee OR Texas OR Utah OR Vermont OR Virginia OR Washington OR West Virginia OR Wisconsin OR Wyoming)
**Scopus**	TITLE-ABS-KEY("Mass gathering*" OR "group gathering*" OR "sporting event*" OR "large assembl*" OR "public assembl*" OR "public gathering*" OR "public meeting*" OR theater* OR auditorium* OR amphitheater* OR Olympic* OR "world cup" OR festival* OR carnival* OR "country fair" OR "county fair" OR "state fair" OR "world fair" OR "world expo" OR "amusement park*" OR "cruise ship*") AND TITLE-ABS-KEY (influenza OR pneumonia OR SARS OR flu OR H1N1 OR H3N2 OR coronavirus OR tuberculosis OR TB OR (lung* w/2 (infection* OR virus*)) OR (respiratory w/2 (infection* OR virus*)) OR MERS OR measles or mumps or "severe acute respiratory syndrome" or legionnaires or legionella OR infectious OR communicable OR illness OR disease*) AND TITLE-ABS-KEY(Transmission* OR outbreak OR epidemi* OR pandemic* OR cluster*)<br><br>AND<br><br>(United States OR USA OR Alabama OR Alaska OR Arizona OR Arkansas OR California OR Colorado OR Connecticut OR Delaware OR Florida OR Georgia OR Hawaii OR Idaho OR Illinois OR Indiana OR Iowa OR Kansas OR Kentucky OR Louisiana OR Maine OR Maryland OR Massachusetts OR Michigan OR Minnesota OR Mississippi OR Missouri OR Montana OR Nebraska OR Nevada OR New Hampshire OR New Jersey OR New Mexico OR New York OR North Carolina OR North Dakota OR Ohio OR Oklahoma OR Oregon OR Pennsylvania OR Rhode Island OR South Carolina OR South Dakota OR Tennessee OR Texas OR Utah OR Vermont OR Virginia OR Washington OR West Virginia OR Wisconsin OR Wyoming)

Search Query: Camps and respiratory disease outbreaks

Database	Strategy
**Medline**	(Camp OR camping).ti,ab,sh.<br><br>AND<br><br>Influenza OR pneumonia OR SARS OR flu OR H1N1 OR H3N2 OR coronavirus OR tuberculosis OR TB OR ((lung* OR respiratory) ADJ2 (infection* OR virus*)) OR MERS OR measles OR mumps OR acute respiratory syndrome OR legionnaires OR legionella OR infectious OR communicable OR illness OR disease*<br><br>AND<br><br>(Transmission* OR outbreak OR epidemi* OR pandemic* OR cluster* OR spread)<br><br>AND<br><br>Exp United States/ OR (United States OR USA OR Alabama OR Alaska OR Arizona OR Arkansas OR California OR Colorado OR Connecticut OR Delaware OR Florida OR Georgia OR Hawaii OR Idaho OR Illinois OR Indiana OR Iowa OR Kansas OR Kentucky OR Louisiana OR Maine OR Maryland OR Massachusetts OR Michigan OR Minnesota OR Mississippi OR Missouri OR Montana OR Nebraska OR Nevada OR New Hampshire OR New Jersey OR New Mexico OR New York OR North Carolina OR North Dakota OR Ohio OR Oklahoma OR Oregon OR Pennsylvania OR Rhode Island OR South Carolina OR South Dakota OR Tennessee OR Texas OR Utah OR Vermont OR Virginia OR Washington OR West Virginia OR Wisconsin OR Wyoming)
**Embase**	(Camp OR camping).ti,ab,sh.<br><br>AND<br><br>Influenza OR pneumonia OR SARS OR flu OR H1N1 OR H3N2 OR coronavirus OR tuberculosis OR TB OR ((lung* OR respiratory) ADJ2 (infection* OR virus*)) OR MERS OR measles OR mumps OR acute respiratory syndrome OR legionnaires OR legionella OR infectious OR communicable OR illness OR disease*<br><br>AND<br><br>(Transmission* OR outbreak OR epidemi* OR pandemic* OR cluster* OR spread)<br><br>AND<br><br>Exp United States/ OR (United States OR USA OR Alabama OR Alaska OR Arizona OR Arkansas OR California OR Colorado OR Connecticut OR Delaware OR Florida OR Georgia OR Hawaii OR Idaho OR Illinois OR Indiana OR Iowa OR Kansas OR Kentucky OR Louisiana OR Maine OR Maryland OR Massachusetts OR Michigan OR Minnesota OR Mississippi OR Missouri OR Montana OR Nebraska OR Nevada OR New Hampshire OR New Jersey OR New Mexico OR New York OR North Carolina OR North Dakota OR Ohio OR Oklahoma OR Oregon OR Pennsylvania OR Rhode Island OR South Carolina OR South Dakota OR Tennessee OR Texas OR Utah OR Vermont OR Virginia OR Washington OR West Virginia OR Wisconsin OR Wyoming)

**Global Health**	(Camp OR camping).ti,ab,sh.<br><br>AND<br><br>Influenza OR pneumonia OR SARS OR flu OR H1N1 OR H3N2 OR coronavirus OR tuberculosis OR TB OR ((lung* OR respiratory) ADJ2 (infection* OR virus*)) OR MERS OR measles OR mumps OR acute respiratory syndrome OR legionnaires OR legionella OR infectious OR communicable OR illness OR disease*<br><br>AND<br><br>(Transmission* OR outbreak OR epidemi* OR pandemic* OR cluster* OR spread)<br><br>AND<br><br>Exp United States/ OR (United States OR USA OR Alabama OR Alaska OR Arizona OR Arkansas OR California OR Colorado OR Connecticut OR Delaware OR Florida OR Georgia OR Hawaii OR Idaho OR Illinois OR Indiana OR Iowa OR Kansas OR Kentucky OR Louisiana OR Maine OR Maryland OR Massachusetts OR Michigan OR Minnesota OR Mississippi OR Missouri OR Montana OR Nebraska OR Nevada OR New Hampshire OR New Jersey OR New Mexico OR New York OR North Carolina OR North Dakota OR Ohio OR Oklahoma OR Oregon OR Pennsylvania OR Rhode Island OR South Carolina OR South Dakota OR Tennessee OR Texas OR Utah OR Vermont OR Virginia OR Washington OR West Virginia OR Wisconsin OR Wyoming)
**CAB Abstracts**	(Camp OR camping).ti,ab,sh.<br><br>AND<br><br>Influenza OR pneumonia OR SARS OR flu OR H1N1 OR H3N2 OR coronavirus OR tuberculosis OR TB OR ((lung* OR respiratory) ADJ2 (infection* OR virus*)) OR MERS OR measles OR mumps OR acute respiratory syndrome OR legionnaires OR legionella OR infectious OR communicable OR illness OR disease*<br><br>AND<br><br>(Transmission* OR outbreak OR epidemi* OR pandemic* OR cluster* OR spread)<br><br>AND<br><br>Exp United States/ OR (United States OR USA OR Alabama OR Alaska OR Arizona OR Arkansas OR California OR Colorado OR Connecticut OR Delaware OR Florida OR Georgia OR Hawaii OR Idaho OR Illinois OR Indiana OR Iowa OR Kansas OR Kentucky OR Louisiana OR Maine OR Maryland OR Massachusetts OR Michigan OR Minnesota OR Mississippi OR Missouri OR Montana OR Nebraska OR Nevada OR New Hampshire OR New Jersey OR New Mexico OR New York OR North Carolina OR North Dakota OR Ohio OR Oklahoma OR Oregon OR Pennsylvania OR Rhode Island OR South Carolina OR South Dakota OR Tennessee OR Texas OR Utah OR Vermont OR Virginia OR Washington OR West Virginia OR Wisconsin OR Wyoming)
**Scopus**	TITLE-ABS-KEY(Camp OR Camping) AND TITLE-ABS-KEY (influenza OR pneumonia OR SARS OR flu OR H1N1 OR H3N2 OR coronavirus OR tuberculosis OR TB OR (lung* w/2 (infection* OR virus*)) OR (respiratory w/2 (infection* OR virus*)) OR MERS OR measles or mumps or "acute respiratory syndrome" or legionnaires or legionella OR infectious OR communicable OR illness OR disease*) AND TITLE-ABS-KEY(Transmission* OR

	outbreak OR epidemi* OR pandemic* OR cluster*) AND TITLE-ABS-KEY (United States OR USA OR Alabama OR Alaska OR Arizona OR Arkansas OR California OR Colorado OR Connecticut OR Delaware OR Florida OR Georgia OR Hawaii OR Idaho OR Illinois OR Indiana OR Iowa OR Kansas OR Kentucky OR Louisiana OR Maine OR Maryland OR Massachusetts OR Michigan OR Minnesota OR Mississippi OR Missouri OR Montana OR Nebraska OR Nevada OR New Hampshire OR New Jersey OR New Mexico OR New York OR North Carolina OR North Dakota OR Ohio OR Oklahoma OR Oregon OR Pennsylvania OR Rhode Island OR South Carolina OR South Dakota OR Tennessee OR Texas OR Utah OR Vermont OR Virginia OR Washington OR West Virginia OR Wisconsin OR Wyoming)
