# Supplementary material for: Mass Gatherings and Respiratory Disease Outbreaks in the United States – Should We Be Worried? Results from a Systematic Literature Review and Analysis of the National Outbreak Reporting System
Source: PLoS One. 2016 Aug 18;11(8):e0160378. doi: 10.1371/journal.pone.0160378 (PMC4990208; doi:10.1371/journal.pone.0160378)
Supplement: S2 File — (PDF) [file pone.0160378.s002.pdf]

Supporting Information 2 – Summary information from articles included in systematic literature review.

1) **Centers for Disease Control and Prevention [16].**
Type of report/study: Outbreak investigation

Mass gathering
Setting: Camp
State: New York
Size of gathering: 541 (including campers and staff)
Approximate dates: June 28 to August 18, 2005

Disease information
Etiology: Mumps (virus)
Mode of transmission: Person-to-Person
Number of case-patients: 31 (including 5 laboratory confirmed), 12 among campers and 19 among staff
Onset date of index case: June 30, 2005 (staff member)
Age of case-patients: 10 to 41 years
Attack rate: 5.7%

Major factors contributing to outbreak
International importation of infectious case-patient (unvaccinated counselor from United Kingdom)
Close social mixing and contact in communal housing (cabins) and during camp activities
Delayed recognition, diagnosis, and reporting

2) **Schaffzin JK, et al. [17] (same outbreak as above).**
Type of report/study: Retrospective cohort and vaccine effectiveness study

Mass gathering
Setting: Camp
State: New York
Size of gathering: 541 (368 youth campers - aged 7 to 15 years, and 173 staff – aged 14 to 65 years)
Approximate dates: June 30 to September 9, 2005 (1- or 2-month overnight summer camp)

Disease information
Etiology: Mumps (virus)
Mode of transmission: Person-to-Person
Number of case-patients: 31 (5 - laboratory confirmed), 12 campers and 19 staff members
Onset date of index case: June 30, 2005
Age of case-patients: 10 to 41 years
Attack rate: 5.7%
Vaccine effectiveness: 92% for two doses of MMR, 80% for 1 dose of MMR

Major factors contributing to outbreak
International importation of infectious case-patient (unvaccinated staff from United Kingdom)
Close social mixing and contact in communal housing (cabins) and during camp activities

Delayed recognition, diagnosis, and reporting

3) **Centers for Disease Control and Prevention (CDC) [18].**
Type of report/study: Case review - Outbreak investigation

Mass gathering
Setting: International sporting event (participants/spectators from Canada, China, Taiwan, Curacao, Japan, Netherlands, Mexico, Saudi Arabia, United States, and Venezuela)
State: Pennsylvania
Size of gathering: 265,000 (including 471 participants and staff)
Approximate dates: August 17 to August 26, 2007

Disease information
Etiology: Measles (virus)
Mode of transmission: Person-to-Person
Number of case-patients: 7 (including index case, all laboratory confirmed), 1 exposed to index case in Japan, 2 co-workers at Detroit airport, and 1 exposed to index case at sporting event, and leading to second generation of 2 cases in Texas.
Onset date of index case: August 15, 2007 (rash onset – infected in Japan)
Hospitalizations: 1
Age of case-patients: 12 to 53 years
Attack rate: Unknown

Major factors contributing to outbreak
International importation of infectious case-patient (unvaccinated participant from Japan)
Transmission during airline-travel and in shared participant housing
Low measles vaccination coverage among participants and staff

4) **Chen TH, et al. [19] (same outbreak as above).**
Type of report/study: Outbreak investigation-case report

Mass gathering
Setting: International sporting event (participants/spectators from Canada, China, Taiwan, Curacao, Japan, Netherlands, Mexico, Saudi Arabia, United States, and Venezuela)
State: Pennsylvania
Size of gathering: 265,000 (471 event participants)
Approximate dates: August 17 to August 26, 2007

Disease information
Etiology: Measles (virus)
Mode of transmission: Person-to-Person
Number of case-patients: 7 (including index case, all laboratory confirmed), 1 exposed to index case in Japan, 2 co-workers at Detroit airport, and 1 exposed to index case at sporting event, and leading to second generation of 2 cases in Texas.

Onset date of index case: August 15, 2007 (rash onset – infected in Japan)
Age of case-patients: 12 to 53 years

Attack rate: Unknown

Major factors contributing to outbreak
International importation of infectious case-patient (unvaccinated participant from Japan)
Transmission during airline-travel and in shared participant housing
Low measles vaccination coverage and/or immunity among participants (41% of 471 participants lacking evidence of measles immunity, including 94 US-resident adults, 19 non-US-resident adults, and 80 non-US children)

5) **Killian ML, et al. [20].**
Type of report/study: Laboratory investigation

Mass gathering
Setting: County Fair
State: Ohio
Size of gathering: Unknown
Approximate dates: August 2007

Disease information
Etiology: Influenza A H1N1 (3SIV)
Mode of transmission: Zoonotic
Number of case-patients: 24 (including 2 laboratory confirmed)
Onset date of index case:
Age of case-patients: Unknown
Attack rate: Unknown

Major factors contributing to outbreak
Close contact between humans and swine at county fair
Both humans and swine ill with identical virus – indicating virus was transmitted from swine to humans or from humans to swine

6) **Shinde V, et al. [21].**
Type of report/study: Case review (Influenza A H1 3SIV viruses)

Mass gathering
Setting: County Fair
State: Ohio (same confirmed case-patients as above), Illinois, and Michigan
Size of gathering: Unknown
Approximate dates: August 2007

Disease information
Etiology: Influenza A H1 (3SIV)
Mode of transmission: (presumed) Zoonotic
Number of case-patients: 4 (All laboratory confirmed)
Onset date of index case:
Age of case-patients: 16 months to 48 years
Attack rate: Unknown

Major factors contributing to outbreak
Direct contact with swine at fair (as exhibitors) in Ohio, where ill swine were present
Visited fair, close proximity or near vicinity of swine (Illinois and Michigan)

7) **Centers for Disease Control and Prevention (CDC) [22].**
Type of report/study: Case review (Oseltamivir-Resistant 2009 Pandemic Influenza A H1N1 cases)

Mass gathering
Setting: Camp
State: North Carolina
Size of gathering: 1,000 (1st session), 650 (2nd session)
Approximate dates: June 14 to August 7, 2009 (2 sessions)

Disease information
Etiology: Pandemic Influenza A H1N1 (virus)
Mode of transmission: Person-to-Person
Number of case-patients: 67 with influenza-like-Illness (including 2 confirmed with Oseltamivir-resistant influenza virus during second session)
Onset date of index case: June 18, 2009
Age of case-patients: Majority (n = 63, including 2 with Oseltamivir resistant infection) were adolescent campers, 4 were camp staff members
Attack rate: Unknown

Major factors contributing to outbreak
Close social mixing and contact in communal housing (cabins) and during camp activities.
Case-patients with Oseltamivir resistant infection were cabin mates – either due to transmission of resistant virus from one case-patient to another, transmission to both case-patients from a 3rd unidentified person with resistant infection, or independent mutation in both case-patients resulting in Oseltamivir resistance (possibly due to sub-therapeutic dose when symptomatic).

8) **Doyle TJ, et al. [23].**
Type of report/study: Cross-sectional survey (of campers returning home)

Mass gathering
Setting: Camp
State: North Carolina
Size of gathering: 700 (212 followed after camp ended and used for estimating number of ill patients and attack rate)
Approximate dates: June 7 to June 13, 2009

Disease information
Etiology: Pandemic Influenza A H1N1 (virus)
Mode of transmission: Person-to-Person
Number of case-patients: 49 cases occurring at camp (including 12 laboratory confirmed cases), 3 additional secondary cases in households following return of ill camper
Onset date of index case: Unknown

Age of case-patients: 10 to 16 years, and adult staff
Attack rate: 23% (among campers and staff), 3.5% (secondary household attack rate – variable exposure time to ill campers)

Major factors contributing to outbreak
Close social mixing and contact in communal housing and during camp activities. Ill campers returned home while still infectious (though after peak infectivity), resulting in secondary transmission

9) **Morrison C, et al. [24].**
Type of report/study: Outbreak investigation and cross-sectional survey (of campers and staff)

Mass gathering
Setting: Camp (for children with hematologic and oncologic conditions – and siblings)
State: Louisiana
Size of gathering: 217 (101 children campers and 116 staff)
Approximate dates: July 26, 2009 (closed on day 5, July 31, due to number of ill campers)

Disease information
Etiology: Pandemic Influenza A H1N1 (virus)
Mode of transmission: Person-to-Person
Number of case-patients: 59 with probable influenza-like-illness (including 10 laboratory confirmed cases – 2 tested at camp, 8 tested at outside facility)
Onset date of index case: July 27, 2009
Age of case-patients: 5 to 69 years
Attack rate: 35.8% (28.7% in healthy campers/staff, 46.9% in campers/staff with underlying condition)

Major factors contributing to outbreak
Close social mixing and contact in communal housing (bunkhouses) and during camp activities. Increased risk of symptomatic infection among campers with hematologic and oncologic condition

10) **Robinson S, et al. [25].**
Type of report/study: Retrospective (web-based) survey

Mass gathering
Setting: Camps
State: Maine
Size of gathering: Range from 10 to 500 campers and 2 to 280 staff per camp
Approximate dates: June 1 to August 30, 2009

Disease information
Etiology: 2009 Pandemic Influenza A H1N1 (virus)
Mode of transmission: Person-to-Person
Number of case-patients: Variable across 19 camps reporting influenza outbreak (≥ 3 confirmed cases)
Onset date of index case: Unknown
Age of case-patients: Unknown (both campers and staff confirmed with influenza)
Attack rate: Variable

Major factors contributing to outbreak
Close social mixing and contact in communal (cabins) and during camp activities
Larger number of campers per session and per cabin associated with greater risk of influenza outbreak.

11) **Kimberlin D, et al. [26].**
Type of report/study: Prospective intervention study (prophylactic course of Oseltamivir and hand hygiene/environmental cleaning on influenza transmission)

Mass gathering
Setting: Camp
State: Alabama
Size of gathering: ~800 (across 4 two-week sessions), 4th session included 246 (171 campers and 75 staff/counselors)
Approximate dates: June - July 2009, 4th session July 19 to July 31, 2009

Disease information
Etiology: Pandemic Influenza A H1N1 (virus)
Mode of transmission: Person-to-Person
Number of case-patients: In 3rd session, 12 cases with influenza-like-illness (including 4 laboratory confirmed cases). In 4th session, 3 cases were laboratory confirmed
Onset date of index case: July 15, 2009 (before start of 4th session)
Age of case-patients: 8 to 14 years in 4th session
Attack rate: 1.8% in 4th session

Major factors contributing to outbreak
Close social mixing and contact in communal housing and during camp activities
Importation of influenza into camp from outside exposures
Intervention of prophylactic use of Oseltamivir with good hand hygiene practices and environmental cleaning may be able to slow transmission in camp setting.

12) **Tsalik EL, et al. [27].**
Type of report/study: Prospective camp-based intervention study

Mass gathering
Setting: University-based camp(s)
State: North Carolina
Size of gathering: 7,906 participants in 73 residential camps (academic, athletic, and single social reunion) across 3 campuses
Approximate dates: May 2 – August 2009 (camps ranging from 3 to 73 days in duration)

Disease information
Etiology: Pandemic Influenza A H1N1 (virus)
Mode of transmission: Person-to-Person
Number of case-patients: Total = 119

- Cluster 1 = 60 (across 3 camps), 4 additional case-patients identified but not epidemiologically linked to cluster (June 15 to July 2). Of 64, 27 were laboratory confirmed.

- Cluster 2 = 47, 8 additional case-patients identified but not linked to other case-patients (July 13 to July 24). Of 55, 40 were laboratory confirmed.

Onset date of index case: Cluster 1 = June 14, Cluster 2 = July 13
Age of case-patients: 9 to 68 years
Attack rate: Among participants in Cluster 1 = 14.9%, among participants in Cluster 2 = 15%

Major factors contributing to outbreak
Close social mixing and contact in communal housing and during camp activities (prolonged close contact in and out of the classroom during academic camps, transmission linked to social interactions outside of class and housing)
Delay in seeking medical attention early after symptom onset
Implementation of infection control activities at may be insufficient to control influenza transmission at large university-based camp

13) **Tsalik EL, et al. [28] (same outbreak as above).**
Type of report/study: Case review (of ILI among participants/staff of university-based camp)

Mass gathering
Setting: University-based camp(s)
State: North Carolina
Size of gathering: 7,906 participants in 73 residential camps (academic, athletic, and single social reunion) across 3 campuses
Approximate dates: May 2 – August 2009 (camps ranging from 3 to 73 days in duration)

Disease information
Etiology: Pandemic Influenza A H1N1 (virus)
Mode of transmission: Person-to-Person
Number of case-patients: Total = 119

- Cluster 1 = 60 (across 3 camps), 4 additional case-patients identified but not epidemiologically linked to cluster (June 15 to July 2). Of 64, 27 were laboratory confirmed.
- Cluster 2 = 47, 8 additional case-patients identified but not linked to other case-patients (July 13 to July 24). Of 55, 40 were laboratory confirmed.

Onset date of index case: Cluster 1 = June 14, Cluster 2 = July 13
Age of case-patients: 9 to 68 years
Attack rate: Among participants in Cluster 1 = 14.9%, among participants in Cluster 2 = 15%

Major factors contributing to outbreak
Close social mixing and contact in communal housing and during camp activities
Inclusion of multiple ILI symptoms in the diagnostic algorithm could help to identify and differentiate influenza infections compared to other causes of ILI

14) **Sugimoto JD, et al. [29].**
Type of report/study: Retrospective survey (to assess affect of age group on susceptibility to symptomatic disease and to estimate transmissibility of pandemic Influenza A H1N1)

Mass gathering
Setting: Camp

State: Washington
Size of gathering: 145 (111 6th-grade students, and 35 teachers and staff)
Approximate dates: April 25 – 30, 2009

Disease information
Etiology: Pandemic Influenza A H1N1 (virus)
Mode of transmission: Person-to-Person
Number of case-patients: 49 camp participants (from 96 campers responding to survey), 11 additional cases among household contacts (among 136 household contacts from 41 households with an ill camper)
Onset date of index case: Unknown
Age of case-patients: Majority of ill campers ≤ 17 years of age
Attack rate: 51% (according to survey respondents) for influenza-like-illness. Secondary household attack rate = 6% for influenza-like-illness (according to survey respondents).

Major factors contributing to outbreak
Close social mixing and contact in communal housing and during camp activities
Intense mixing between children (often at greater risk of influenza infection) in congregate settings such as camps provide greater opportunities for transmission than within households (number of susceptible children will often be depleted due infection at other settings)

15) **Centers for Disease Control and Prevention [30].**
Type of report/study: Outbreak report – case review

Mass gathering
Setting: Camp
State: New York
Size of gathering: 400
Approximate dates: June - August 2009

Disease information
Etiology: Mumps (virus)
Mode of transmission: Person-to-Person
Number of case-patients: 25 at camp, 79 additional cases in community following end of camp
Onset date of index case: June 28
Age of case-patients: 9 to 30 years at camp, 8 months to 84 in community
Attack rate: 6% at camp

Major factors contributing to outbreak
International importation of infectious case-patient 11-year old from United Kingdom
Close social mixing and contact in communal housing (cabins) and during camp activities
Waning or lack of immunity for mumps: Among 24 camp participants with known vaccination status, 20 (83%) were age-appropriately vaccinated, one (4%) was partially vaccinated, and three (13%) were unvaccinated.

16) **Centers for Disease Control and Prevention (same outbreak as above - update) [31].**
Type of report/study: Outbreak report-case review

Mass gathering
Setting: Camp
State: New York
Size of gathering: 400
Approximate dates: June - August 2009

Disease information
Etiology: Mumps (virus)
Mode of transmission: Person-to-Person
Number of case-patients: 25 at camp, 1,521 total between June 2009 and January 2010
Onset date of index case: June 28, 2009
Age of case-patients: 3 months to 90 years (includes camps participants/staff and subsequent cases from community)
Attack rate: Unknown

Major factors contributing to outbreak
International importation of infectious case-patient 11-year old from United Kingdom
Close social mixing and contact in communal housing (cabins) and during camp activities
Prolonged close contact in other congregate settings such as school and larger mean household size (in impacted communities)
Waning of lack of mumps immunity

17) **Cox CH, et al. [32].**
Type of report/study: Case report

Mass gathering
Setting: Fair
State: Kansas
Size of gathering: Unknown
Approximate dates: July 2009

Disease information
Etiology: Influenza A H3N2 (SIV)
Mode of transmission: Zoonotic
Number of case-patients: 1
Onset date of index case: July 28, 2009
Age of case-patients: 12 years
Attack rate: Unknown

Major factors contributing to outbreak
Close contact swine at county fair

18) **Centers for Disease Control and Prevention [33].**
Type of report/study: Case report

Mass gathering
Setting: Fair
State: Pennsylvania
Size of gathering: Unknown
Approximate dates: August 2011

Disease information
Etiology: Influenza A H3N2v
Mode of transmission: Zoonotic
Number of case-patients: 1
Onset date of index case: August 20, 2011
Age of case-patients: < 5 years
Attack rate: Unknown

Major factors contributing to outbreak
Close contact with swine at county fair

19) **Wong KK, et al. [34].**
Type of report/study: Outbreak investigation and retrospective cohort study

Mass gathering
Setting: Fair
State: Pennsylvania
Size of gathering: 70,000
Approximate dates: August 2011

Disease information
Etiology: Influenza A H3N2v
Mode of transmission: Zoonotic
Number of case-patients: 89 (3 confirmed, 4 probable, and 82 suspected)
Onset date of index case: Unknown
Age of case-patients: ≤ 13 years (confirmed and probable cases)
Attack rate: Unknown

Major factors contributing to outbreak
Close contact with swine at county fair
Co-circulation of human and swine influenza viruses at agricultural fairs providing opportunities for emergence of novel strains

20) **Centers for Disease Control and Prevention [35].**
Type of report/study: Outbreak report-case review

Mass gathering
Setting: Fair
State: Indiana
Size of gathering: Unknown
Approximate dates: July 8 to July 14, 2012

Disease information
Etiology: Influenza A H3N2v
Mode of transmission: Zoonotic
Number of case-patients: 4 (all laboratory confirmed)
Onset date of index case: July 12, 2012
Age of case-patients: Unknown
Attack rate: Unknown

Major factors contributing to outbreak
Close contact with swine as exhibitors or family members of exhibitors

21) **Jhung MA, et al. [36].**
Type of report/study: Outbreak investigation-summary case report

Mass gathering
Setting: Fairs
State: Multiple – Illinois, Indiana, Maryland, Michigan, Minnesota, Ohio, Pennsylvania, and Wisconsin
Size of gathering: 22,000 to 1.7 million (estimates only)
Approximate dates: Fairs held during summer and fall 2012

Disease information
Etiology: Influenza A H3N2v
Mode of transmission: Zoonotic (limited person-to-person transmission)
Number of case-patients: Ranging from 1 to 73 across 38 fairs reporting cases (15 additional cases through person-to-person transmission)
Onset date of index case: July 9, 2012
Age of case-patients: 3 months to 74 years
Attack rate: Unknown

Major factors contributing to outbreak
Efficient transmission of H3N2v among swine
Direct or indirect human contact with swine at agricultural fairs
Presence of M gene from pdm09 H1N1 identified in H3N2v viruses, possibly facilitating transmission
